# Long-term health related quality of life in total knee arthroplasty

**DOI:** 10.1186/s12891-023-06399-6

**Published:** 2023-04-25

**Authors:** Marta González-Sáenz-de-Tejada, Jose M. Quintana, Juan C. Arenaza, Jesús R. Azcarate-Garitano, Pedro M. Esnaola-Guisasola, Isidoro García-Sánchez, Alejandro Baguer-Antonio, Amaia Bilbao-González

**Affiliations:** 1 Kronikgune Institute for Health Services Research, Barakaldo, Spain; 2 Osakidetza Basque Health Service, Research Unit, Basurto University Hospital, Bilbao, Spain; 3 Health Services Research on Chronic Patients Network (REDISSEC), Bilbao, Spain; 4 Network for Research on Chronicity, Primary Care, and Health Promotion (RICAPPS), Bilbao, Spain; 5 Hospital Universitario de Basurto. Unidad de Investigación, Jado 4º Planta. Avda. Montevideo 18, Bilbao, 48013 Bizkaia Spain; 6 Osakidetza Basque Health Service, Research Unit, Galdakao-Usansolo University Hospital, Galdakao, Bizkaia Spain; 7 Osakidetza Basque Health Service, Traumatology and Orthopedic Surgery Service, Basurto University Hospital, Bilbao, Bizkaia Spain; 8 Osakidetza Basque Health Service, Mendaro Hospital, Traumatology and Orthopedic Surgery Service, Mendaro, Spain; 9 Osakidetza Basque Health Service, Traumatology and Orthopedic Surgery Service, Donostia University Hospital, Donostia, Gipuzkoa Spain; 10 Osakidetza Basque Health Service, Galdakao-Usansolo University Hospital, Traumatology and Orthopedic Surgery Service, Galdakao, Bizkaia Spain

**Keywords:** Health Related Quality of Life, Osteoarthritis, Total knee arthroplasty, Long-term, Minimal clinically important differences

## Abstract

**Background:**

To analyze evolution and factors related with greater gains in Health Related Quality of Life (HRQOL) and with a greater probability of exceed their corresponding minimal clinically important differences (MCID) in patients with Osteoarthritis of the knee, undergoing total knee arthroplasty (TKA) at long-term.

**Methods:**

Data were obtained from two previously recruited multicenter cohorts of patients who underwent TKA in the Basque Country. Patients were follow-up at 6 months and 10 years after surgery. Patients completed specific and generic HRQOL questionnaires plus sociodemographic, and clinical data at 10 years. Associations were analysed using linear and logistic regression models.

**Results:**

A total of 471 patients responded at 10-year follow-up. The multivariable analysis showed that low preoperative HRQOL scores, higher age, higher BMI, some comorbidities and readmissions at 6 months were associated with less gains in HRQOL. Apart from aforementioned, to have a peripheral vascular disease (odd ratio 0.49 (95% CI, 0.24–0.99)), complications (odd ratio 0.31 (95% CI, 0.11–0.91)), and readmissions within 6 months of discharge (odd ratio 2.12 (95% CI, 1.18–3.80)) were associated with a lower probability of exceeding the MCID. The effect sizes (ESs) of changes from baseline to 6 months (range, 1.20–1.96) and to 10 years (range, 1.54–1.99) were large in all dimensions, nevertheless the ESs from 6 months to 10 years were not appreciable for pain (ES = 0.03) or stiffness (ES = 0.09), and small for function (ES = 0.30).

**Conclusions:**

Low preoperative HRQOL scores, to be elderly, severe obesity, the presence of some comorbidities -depression and rheumatology disease-, having readmissions or complications and not having rehabilitation of discharge, are good predictors of long-term lower gains in HRQOL. Some other non-registered parameters of the follow-up may also influence those outcomes.

**Key Indexing Terms (MeSH terms):**

Health-Related Quality of Life, Knee Arthroplasty, Total, Osteoarthritis

**Supplementary Information:**

The online version contains supplementary material available at 10.1186/s12891-023-06399-6.

## Background

Osteoarthritis (OA) is a chronic disease and one of the major causes of pain and disability in developed countries. Knee OA is a common condition that require regular follow-up, medical therapy, and potentially expensive treatments [1], and the prevalence in Spain, is approximately 13.8% [2]. The main symptoms of OA are pain and loss of physical function [3], both have a negative impact on patients’ health-related quality of life (HRQOL) [4].

In addition to clinical factors, there is a growing interest in HRQOL. Disease-specific or generic HRQOL instruments are widely used to evaluate health outcomes in clinical practice and, increasingly, to assess improvements in pain and function after knee surgery and evaluate the success of total knee arthroplasty (TKA) [5–7].

For knee OA patients that do not respond to medical treatment, TKA is the most effective surgical procedure to reduce pain, increase mobility and improve patients’ HRQOL [8–10], with demonstrably effective treatment in the short term follow-up. Although there is also a substantial body of literature on long-term outcomes, most of the studies have a short and mid-term follow-up(4;11;12).

A recent review [12] of possible patient-related factors and how they affect HRQOL in the mid- and long-term after TKA suggests that little is known about the determining factors in the long-term. To our knowledge, only a very few studies have examined HRQOL over 10 years of follow-up [6, 13, 14]. Further information on determinants of long-term outcomes after TKA might help to prioritize patients for surgery and meet their needs, optimizing outcomes of the procedure.

On the other hand, all of these studies analyzing HRQOL in the long term have been based on questionnaire scores. To facilitate the interpretation of changes in individual patient scores, researchers have developed the concept of minimal clinically important difference (MCID) [15]. In addition to seeing what factors influence scale scores, it is also useful to analyze what influences whether a clinical benefit is achieved in the long term, as measured by the MCID .

The objective of this study was to assess changes in the HRQOL of patients10 years after TKA, comparing it to the HRQOL of the general population, analyze which baseline factors were associated with the greatest gains in HRQOL in the long-term and with a greater probability of exceeding the MCID for each HRQOL instrument.

## Methods

### Study population

This prospective study involved two previously recruited cohorts of patients who underwent TKA in one of nine hospitals in the Basque Country [16, 17]. Consecutive patients scheduled to undergo TKA for primary knee OA were eligible for the study. One cohort was recruited between September 2002 and September 2004 and the other between April 2005 and December 2006. All the participants gave written informed consent.

To be included in the cohorts, patients had to meet the following criteria: be aged over 18 years, be undergoing TKA, have a diagnosis of primary OA and agree to participate in the study. The exclusion criteria were: having a terminal illness, or psychiatric or sensory disturbances that might prevent them from answering the questionnaires, or failing to provide informed consent. For this long-term follow-up study, the additional exclusion criteria were: patients dying during the follow-up period or being over 90 years of age at baseline. The information about deaths was obtained from the Spanish National Death Index. The institutional review board “Basque Research Ethics Committee on Drug Research (CEIm-E)” PI2014049, of the participating hospitals approved the study.

Data of the general population was obtained from the 2007 Basque Health Survey [18].

### Measurements

All patients were sent a letter informing them about the study and asking for their voluntary participation. We mailed questionnaires to each patient before surgery, and 6 months and 10 years after surgery. Reminder letters were sent 15 days after each mailing to patients who had not replied promptly and an attempt was made to contact them by telephone if they had still not answered 15 days later. Any patients from whom no response had been received after another 15 days were sent the questionnaire again.

The baseline questionnaire included the Western Ontario and McMaster Universities Arthritis Index (WOMAC), plus questions requesting clinical and sociodemographic information. At 6 months, the WOMAC questionnaire was sent again. The 10-year mailing included the 36-item Short Form Health Survey (SF-36) in addition to the questionnaires sent previously. Furthermore, in the 6-moths follow-up, we included transition questions about any improvement or deterioration in pain, stiffness or function after TKA (“Compared to before surgery, how would you rate your pain/stiffness/function in the same knee?”). The five responses were “a great deal better”, “somewhat better”, “equal”, “somewhat worse” and “a great deal worse”. Clinical data were collected by trained personnel from the patients’ medical records.

The WOMAC, a questionnaire specific to hip and knee OA, is a multidimensional scale consisting of 24 items grouped into three subscales: pain (5 items), stiffness (2 items), and function (17 items). We used the Likert version of the WOMAC with five response levels for each item, representing different degrees of intensity that were scored from 0 (none) to 4 (extreme). The data are standardized, generating scores for each dimension ranging from 0 (best health status) to 100 (worst) [19]. The WOMAC has been translated into Spanish and validated in a population in Spain [20, 21].

The SF-36 is a generic questionnaire that includes 36 items grouped into eight health concepts: physical function, physical role, bodily pain, general health, vitality, social function, emotional role and mental health. The SF-36 generates two summary scores, the physical and mental component summary scores [22]. Scores vary from 0 (worst health status) to 100 (best health status). We used a Spanish version of the SF-36 [23].

### Statistical analysis

Descriptive statistics included frequency tables for categorical variables and mean and standard deviation (SD) for continuous variables. Patient characteristics were compared between responders and non-responders to the long-term follow-up questionnaires. Chi-square or Fisher’s exact test were performed for the comparison of categorical variables and Student’s t-test or nonparametric Wilcoxon tests for continuous variables.

Changes in WOMAC scores were explored by comparing WOMAC preoperative, 6-month and long-term follow-up scores using paired t-tests. Further, standardized effect sizes (ES) were estimated to study the magnitude of change, using Cohen’s guidelines for the interpretation [24].

The long-term follow-up SF-36 scores were compared with scores in the general population using the normalized scores. To do so, each SF-36 score was first standardized using the mean and SDs according to sex and age group obtained from the 2007 Basque Health Survey [18]. Then, standardized scores were transformed to norm-based (mean = 50, SD = 10) scores, as suggested by the authors of the questionnaire [25].

To study the effect of baseline factors on changes in HRQoL from baseline to long-term follow-up, the general linear models were used. In addition to baseline factors, we have also considered the knee reintervention during the follow-up as a possible confounding variable in the models. First, univariable analyses were performed to study the effect of each factor individually, but adjusting for the corresponding preoperative WOMAC scores. The dependent variables were changes in WOMAC pain, stiffness or function scores, calculated by subtracting the follow-up from the preoperative scores, with a positive value indicating an improvement. Then, multivariable analyses were performed to analyze the effect of baseline factors and reintervention of the knee jointly on changes in WOMAC scores. The factors with a significance of p < 0.20 in the univariable analyses were considered potential independent variables in the multivariable general linear models. In the final models, only factors with p < 0.05 were retained, but all models were adjusted for age and sex. Possible interactions between variables were also examined. R^2^ was calculated to assess the predictive accuracy of the models. Lastly, based on the final models, multilevel analyses were performed with linear mixed models including a hospital-level random effect, to account for variation between hospitals.

Regarding the MCID, we first estimated the MCID for each WOMAC score, based on the transition questions at 6-months follow-up. That is, the MCID was estimated by calculating the mean change scores from before to 6-months after surgery in patients whose response to the transition question was “somewhat better” [26]. Based on these cut-off points, we analyzed the percentage of patients exceeding the MCID for each WOMAC dimension at 6 months and 10 years of follow-up. Then, to study the effect of baseline factors on the probability of exceeding the MCID at 10 years of follow-up, the univariable and multivariable logistic regression models were used using the same procedure as explained above. The predictive accuracy of the models was assessed by the area under the receiver operating characteristic curve (AUC) [27]. Lastly, based on the final models, multilevel analyses were performed with generalized linear mixed models including a hospital-level random effect.

All effects were considered significant at p < 0.05. All statistical analyses were performed using SAS for Windows statistical software, version 9.4 (SAS Inc., Cary, NC).

## Results

Of the total sample of 1107 patients obtained from the previous two cohorts, 376 did not meet the selection criteria, and of these, 246 having died during follow-up. We have included an additional table (Additional Table 1) with the comparison of baseline characteristics between patients who have died during follow-up and those included in the analysis. We found that those who died more were older, female, had more comorbidities, more surgical risk and less rehabilitation from hospital discharge. Of the remaining 731 patients, 471 (64.43%) responded to the questionnaires at the 10-year follow-up. The mean follow-up was 10.11 years (SD = 0.76). Data on descriptive variables are summarized in Table 1. We found that responders had a mean age of 69.73 years (SD = 6.26), 75.37% were female, 87.83% had social support, 62.91% were married, 82.17% lived accompanied, 43.57% were housekeeper and 39.87% were retired.Further, responders had slightly better (mean = 59.76, SD = 16.77 vs. mean = 63.33, SD = 18.21) baseline WOMAC function scores than patients who did not respond to the questionnaires (non-responders) (p = 0.008). Data related to reinterventions are described in the Additional Table 2; 9.25% of patients had a reintervention, the main reason was aseptic loosening/mobilization (37.21%) and the mean time from intervention to reintervention was 3.10 years (SD 3.03).

**Table 1:** Baseline characteristics of responders and non-responders to the long-term follow-up

Variables	Responders (n = 471)	Non-responders (n = 260)	p-value
**Age** in years: mean (SD)	69.73 (6.26)	71.44 (6.69)	0.001
**Age categorized**: n (%)			< 0.0001
≤ 65	102 (22.67)	31 (12.35)	
65–75	263 (58.44)	133 (52.99)	
≥ 75	85 (18.89)	87 (34.66)	
**Gender**: female: n (%)	355 (75.37)	196 (75.38)	0.997
**BMI**: mean (SD)	30.22 (4.34)	30.30 (4.57)	0.816
**BMI categorized**: n (%)			0.444
BMI < 25	38 (8.78)	27 (11.49)	
25 ≤ BMI < 30	181 (41.80)	90 (38.30)	
30 ≤ BMI < 35	159 (36.72)	80 (34.04)	
BMI ≥ 35	55 (12.60)	38 (16.17)	
**Having social support**: n (%)	397 (87.83)	203 (81.20)	0.017
**Civil status**: n (%)			0.246
Married	290 (62.91)	142 (56.57)	
Partner	25 (5.42)	10 (3.98)	
Divorced	5 (1.08)	5 (1.99)	
Widowed	128 (27.77)	83 (33.07)	
Single	13 (2.82)	11 (4.38)	
**Current situation**: n (%)			0.044
I live alone	70 (15.22)	51 (20.24)	
I live accompanied	378 (82.17)	189 (75.00)	
Residence	0 (0.00)	2 (0.79)	
Others	12 (2.61)	10 (3.97)	
**Employment situation**: n (%)			0.252
Active	9 (1.96)	3 (1.21)	
Temporary inability to work	16 (3.49)	3 (1.21)	
Housekeeper	200 (43.57)	98 (39.52)	
Unemployed	4 (0.87)	5 (2.02)	
Retired	183 (39.87)	107 (43.15)	
Early retirement	42 (9.15)	27 (10.89)	
Others	5 (1.09)	5 (2.02)	
**Comorbidity**: n (%)			
Myocardial infarction	14 (3.14)	10 (4.05)	0.531
Congestive heart disease	17 (3.80)	11 (4.45)	0.677
Hypertension	243 (54.61)	141 (57.09)	0.530
Peripheral vascular disease	62 (13.90)	27 (10.93)	0.263
Chronic pulmonary disease	38 (8.52)	25 (10.12)	0.483
Ulcer disease	17 (3.81)	14 (5.67)	0.258
Mild liver disease	3 (0.67)	0 (0)	0.556
Moderate or severe chronic kidney disease	2 (0.45)	2 (0.81)	0.619
Diabetes	52 (11.69)	33 (13.36)	0.520
Cancer Tumour	6 (1.35)	8 (3.24)	0.098
Cerebrovascular disease	13 (2.91)	10 (4.05)	0.425
Back pain (backache)	44 (9.87)	25 (10.12)	0.914
Rheumatologic disease	38 (8.52)	21 (8.54)	0.994
Connective tissue disease	2 (0.45)	2 (0.81)	0.619
Depression	36 (8.07)	34 (13.77)	0.017
**Other pathologies**: n (%)			
Back	79 (19.13)	58 (25.22)	0.071
Homolateral hip	17 (4.17)	14 (6.17)	0.262
Contraletral hip	23 (5.62)	11 (4.85)	0.676
Contralateral knee	205 (49.52)	105 (45.85)	0.373
Upper limbs	20 (4.91)	16 (7.08)	0.260
**OA severity** (Ahlbäck scale): n (%)			0.671
Mild	6 (1.79)	3 (1.72)	
Moderate	75 (22.32)	45 (25.86)	
Severe	255 (75.89)	126 (72.41)	
**Surgical risk** (ASA): n (%)			0.702
Low (ASA I, II, III)	426 (99.07)	231 (98.72)	
High (ASA IV)	4 (0.93)	3 (1.28)	
**Intraoperative complications**: n (%)	8 (1.87)	2 (0.85)	0.507
**Postoperative complications**: n (%)	61 (14.49)	35 (15.09)	0.837
**Readmissions (6 months)**: n (%)	62 (14.00)	43 (17.84)	0.183
**Days in hospital**: median (IQR)	12 (10–15)	11 (9–15)	0.252
**Complications from hospital discharge**: n (%)	37 (8.55)	22 (9.28)	0.747
**Rehabilitation from hospital discharge**: n (%)	157 (41.10)	81 (39.51)	0.709
**WOMAC at pre-intervention**: mean (SD)			
Pain	55.72 (17.12)	56.95 (19.46)	0.398
Stiffness	56.92 (23.95)	58.51 (25.36)	0.403
Function	59.76 (16.77)	63.33 (18.21)	0.008

Abbreviations: SD, Standard deviation; IQR, Interquartile range; OA, Osteoarthritis; BMI, Body Mass Index; WOMAC, Western Ontario and McMaster Universities Osteoarthritis Index (0, best; 100, worst)

**Table 2:** Factors associated with changes in HRQOL 10 years after TKA by general linear models

	Change in WOMAC^*^					
	Pain		Stiffness		Function	
	*β*	*p* *value*	*β*	*p* *value*	*β*	*p* *value*
Age (as continuous)	-0.34	**0.036**	-0.50	**0.008**	-0.76	**< 0.001**
Age categorized						
≤ 65	-ref-	-ref-	-ref-	-ref-	-ref-	-ref-
65–75	-2.22	0.373	-0.74	0.798	-2.76	0.312
≥ 75	-2.26	0.479	-5.72	0.123	-9.80	**0.005**
Gender						
Female	-ref-	-ref-	-ref-	-ref-	-ref-	-ref-
Male	4.37	0.058	2.84	0.301	5.89	**0.020**
BMI (as continuous)	-0.85	**0.0004**	-0.82	**0.004**	-0.87	**0.001**
BMI categorized						
BMI < 25	-ref-	-ref-	-ref-	-ref-	-ref-	-ref-
25 ≤ BMI < 30	-3.56	0.342	-1.48	0.746	-1.17	0.780
30 ≤ BMI < 35	-6.88	**0.070**	-3.97	0.388	-5.96	0.159
BMI ≥ 35	-12.87	**0.004**	-11.11	**0.038**	-12.23	**0.014**
Having social support	3.09	0.323	6.39	**0.081**	0.71	0.839
Civil status categorized						
Married or Partner	-3.06	0.151	-1.60	0.528	-0.08	0.975
Divorced, widowed or single	-ref-	-ref-	-ref-	-ref-	-ref-	-ref-
Comorbidity						
Myocardial infarction	0.25	0.965	-12	0.080	-3.83	0.566
Congestive heart disease	-2.81	0.610	3.14	0.637	2.91	0.630
Hypertension	-2.07	0.308	-2.99	0.222	-6.15	**0.007**
Peripheral vascular disease	-1.11	0.701	-1.95	0.577	-2.91	0.371
Chronic pulmonary disease	2.68	0.460	5.48	0.208	2.61	0.522
Ulcer disease	-6.75	0.206	-6.91	0.269	-2.51	0.668
Diabetes	-2.37	0.451	-0.48	0.900	-5.02	0.159
Cancer Tumour	-0.18	0.983	4.60	0.658	2.94	0.763
Cerebrovascular disease	-6.79	0.269	-8.86	0.212	-7.97	0.249
Back pain (backache)	-5.31	0.112	-3.09	0.447	-3.10	0.411
Rheumatologic disease	-8.99	**0.013**	-5.96	0.170	-6.37	0.121
Depression	-10.24	**0.006**	-11.17	**0.012**	-15.43	**< 0.001**
Other pathologies						
Back	-3.96	0.133	-0.34	0.915	-3.57	0.230
Homolateral hip	-7.34	0.170	-6.29	0.311	-6.17	0.308
Contraletral hip	-1.88	0.683	-1.95	0.718	-3.21	0.536
Contralateral knee	-3.31	0.114	-5.46	**0.029**	-2.63	0.265
Upper limbs	-6.34	0.187	-9.94	0.084	-5.91	0.275
OA severity (Ahlbäck scale)						
Mild or moderate	-ref-	-ref-	-ref-	-ref-	-ref-	-ref-
Severe	3.46	0.208	2.055	0.515	3.97	0.200
Days in hospital	-0.23	0.402	-0.89	**0.005**	-0.46	0.134
Postoperative complications	3.98	0.178	3.26	0.357	4.84	0.146
Readmissions (6 months)	-1.42	0.629	-7.05	**0.045**	-4.92	0.131
Complications from hospital discharge	-5.72	0.120	-9.51	**0.032**	-7.37	0.074
Rehabilitation from hospital discharge	1.54	0.482	1.65	0.527	0.64	0.795
Reintervention during the follow-up	-7.63	**0.024**	-8.56	**0.034**	-9.70	**0.010**

Abbreviations: WOMAC, Western Ontario and McMaster Universities Osteoarthritis Index (0, best; 100, worst); BMI = Body Mass Index; OA = Osteoarthritis; ref: reference group

This general linear models have been adjusted for the corresponding WOMAC baseline scores

^*^Changes are calculated so that a positive value indicates improvement and a negative value, worsening in all questionnaires

Regarding changes in WOMAC scores from baseline to the long-term follow-up, patients’ scores showed statistically significant improvements to both follow-ups (p < 0.0001). The improvements observed at 6 months ( around 30 points) were maintained at 10 years, and changes from 6 months to 10 years were not significant (around 1), except for the function dimension (-5.29;p < 0.0001), in which scores indicated worsening. Nevertheless, while the ES of changes from baseline to 6 months (range, 1.20–1.96) and to 10 years (range, 1.54–1.99) were large in all dimensions, the ES from 6 months to 10 years were not appreciable for pain (ES = 0.03) or stiffness (ES = 0.09), and small for function (ES = 0.30). The evolution of the WOMAC dimensions from pre-intervention, to 6-months and 10-years follow-up was shown in Additional Fig. 1.

**Fig. 1:**
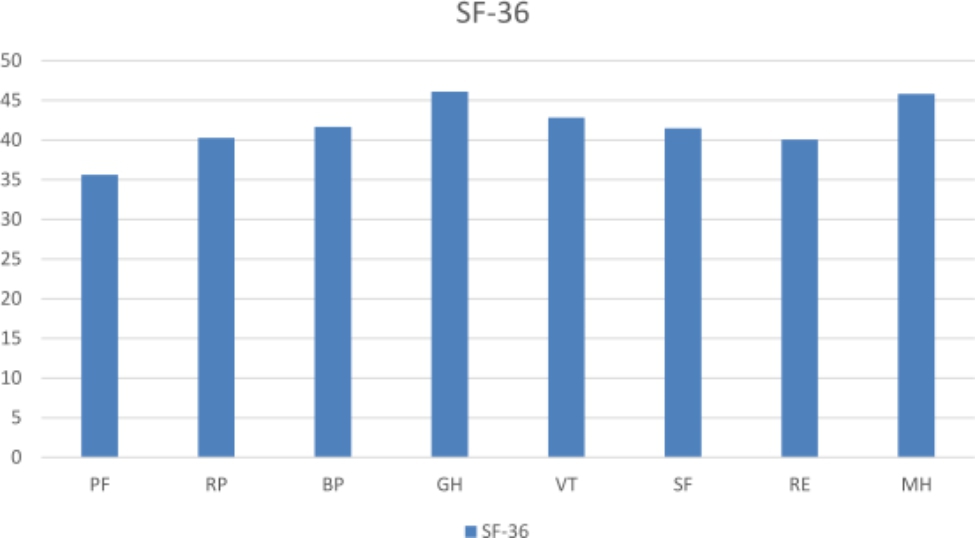
**Comparison of the SF-36 scores with the general population using the normalized scores.** That is, SF-36 scores were standardized according to sex and age of the general population, and transformed to norm-based, considering a mean of 50 SF-36 domains: PF: physical function; RP: role physical; BP: bodily pain; GH: general health; SF: social function; RE: role emotional; VT: vitality; MH: mental health

Regarding the MCID, the cut-off points for changes in score were 29.88 for pain, 26.76 for stiffness and 32.72 for function. The numbers of patients exceeding the MCID for pain, stiffness and function were respectively: 271 (59.04%), 201 (43.98%) and 211 (46.07%) at 6 months; and 281 (62.03%), 207 (45.20%) and 179 (39.17%) at 10 years.

The comparison of HRQOL in patients with TKA and the general population is shown in Fig. 1. The reference score for the general population was 50 points. HRQOL scores for the 10-year cohort were lower than those for the general population after adjusting for age and sex, scores not reaching 50 for any of the SF-36 dimensions.

Table 2 shows the univariable analysis for the relationships between baseline characteristics and changes in WOMAC dimensions at 10 years. The multivariable analysis (Table 3) showed that preoperative WOMAC scores was related to changes in all WOMAC dimensions (p < 0.0001). A higher WOMAC score at baseline was associated with greater improvements at 10-years follow-up. Furthermore, older age, higher BMI and being depressed at baseline were associated with smaller gains at the 10-year follow-up in all WOMAC dimensions, while with having a rheumatic disease was negatively associated with improvements in pain and readmissions at 6 months with improvements in function. The percentage of variance explained by the models ranged from 22.8 to 39.4%. Multilevel analyses revealed that the hospital-level random effect did not modify the results.

**Table 3:** Factors associated jointly with change in HRQOL 10 years after TKA: multivariable analysis

Factors	Change in WOMAC^*^						
	Pain			Stiffness		Function	
	β	*p* *value*	β		*p* *value*	β	*p* *value*
Baseline WOMAC	0.732	< 0.0001	0.77		< 0.0001	0.57	< 0.0001
Age (continuous)	-0.38	0.031	-0.58		0.005	-0.82	< 0.0001
BMI categorized							
BMI < 30	-ref-	-ref-	-ref-		-ref-	-ref-	-ref-
30 ≤ BMI < 35	-4.67	0.042	-4.46		0.104	-6.82	0.008
BMI ≥ 35	-9.14	0.006	-9.65		0.014	-11.43	0.002
Comorbidity							
Depression	-9.66	0.012	-11.32		0.013	-11.77	0.008
Rheumatologic disease	-8.19	0.025					
Readmissions (6 months)						-7.29	0.037
**R** ^**2**^	0.314			0.394		0.228	

Abbreviations: BMI, Body Mass Index; WOMAC, Westen Ontario and McMaster Universities Osteoarthritis Index (0, best; 100, worst); ref: reference group

^*^Changes are calculated so that a positive value indicates improvement and a negative value, worsening in all domains

These general linear models have been adjusted for age and gender and they only appear in the table if some of them were statistically significant

Table 4 shows the univariable analyses of factors associated with change in WOMAC at 10 years exceeding the MCID adjusting for baseline WOMAC scores. The multivariable analyses are presented in Table 5. The variables significantly associated with a lower probability of exceeding the MCID were: (1) for pain, having a lower preoperative pain score (p < 0.001), and being diagnosed with depression (p = 0.036) or a rheumatic disease (p = 0.011); (2) for stiffness, having a low preoperative stiffness score (p < 0.001), BMI ≥ 25 kg/m^2^ (p < 0.05), depression (p = 0.015), and complications (p = 0.032) and not having rehabilitation after discharge (p = 0.012); and (3) for function, having a low preoperative function score (p < 0.001), being older (p = 0.001) and having a high BMI (p < 0.05), peripheral vascular disease (p = 0.048) or depression (p = 0.003) and readmissions within 6 months (p = 0.035). The AUC of the models ranged from 0.74 to 0.85. Multilevel analyses revealed that the hospital-level random effect did not modify the results.

**Table 4:** Factors associated with change in WOMAC at 10 years exceeding the minimal clinically important difference

Factors	Change in WOMAC exceeding MCID^*^					
	Pain		Stiffness		Function	
	Odds ratio^**^ (95% CI)	*p* *value*	Odds ratio (95% CI)	*p* *value*	Odds ratio (95% CI)	*p* *value*
Age (as continuous)	1 (0.97–1.04)	0.849	0.99 (0.96–1.03)	0.608	0.95 (0.92–0.98)	**0.003**
Age categorized						
≤ 65	-ref-	-ref-	-ref-	-ref-	-ref-	-ref-
65–75	1.06 (0.62–1.80)	0.842	1.20 (0.68–2.11)	0.525	0.77 (0.47–1.28)	0.318
≥ 75	1.82 (0.92–3.64)	0.088	1.28 (0.62–2.63)	0.500	0.60 (0.31–1.15)	0.125
Gender						
Female	-ref-	-ref-	-ref-	-ref-	-ref-	-ref-
Male	1.06 (0.66–1.71)	0.810	0.82 (0.48–1.38)	0.446	1.60 (1-2.54)	**0.048**
BMI (as continuous)	0.93 (0.88–0.98)	**0.004**	0.93 (0.88–0.98)	**0.009**	0.93 (0.89–0.98)	**0.006**
BMI categorized						
BMI < 25	-ref-	-ref-	-ref-	-ref-	-ref-	-ref-
25 ≤ BMI < 30	0.61 (0.25–1.47)	0.271	0.48 (0.20–1.14)	0.098	0.59 (0.27–1.28)	0.184
30 ≤ BMI < 35	0.35 (0.14–0.86)	**0.022**	0.40 (0.17–0.95)	**0.038**	0.46 (0.21–1.02)	0.055
BMI ≥ 35	0.39 (0.14–1.06)	0.065	0.29 (0.11–0.81)	**0.018**	0.27 (0.11–0.69)	**0.006**
Social support	1.17 (0.61–2.25)	0.631	1.67 (0.81–3.42)	0.164	1.08 (0.57–2.06)	0.809
Civil status categorized						
Married or Partner	0.60 (0.38–0.95)	**0.028**	0.70 (0.43–1.12)	0.134	1.17 (0.75–1.80)	0.492
Divorced, widowed or single	-ref-	-ref-	-ref-	-ref-	-ref-	-ref-
Comorbidity						
Myocardial infarction	0.87 (0.28–2.76)	0.817	0.39 (0.10–1.59)	0.189	1.09 (0.34–3.49)	0.890
Congestive heart disease	0.84 (0.27–2.67)	0.767	1.83 (0.53–6.26)	0.337	1.64 (0.56–4.78)	0.365
Hypertension	0.67 (0.44–1.02)	0.062	0.89 (0.57–1.40)	0.613	0.63 (0.42–0.95)	**0.028**
Peripheral vascular disease	0.68 (0.37–1.24)	0.208	0.70 (0.36–1.34)	0.277	0.47 (0.25–0.88)	**0.017**
Chronic pulmonary disease	0.95 (0.44–2.05)	0.896	0.96 (0.42–2.20)	0.931	1.30 (0.63–2.68)	0.478
Ulcer disease	0.64 (0.22–1.88)	0.418	0.39 (0.12–1.31)	0.127	0.68 (0.24–1.95)	0.469
Diabetes	0.76 (0.39–1.46)	0.404	0.60 (0.29–1.24)	0.166	0.72 (0.38–1.37)	0.317
Cancer Tumour	0.64 (0.11–3.65)	0.619	0.46 (0.05–4.69)	0.514	1.48 (0.26–8.38)	0.658
Cerebrovascular disease	0.81 (0.24–2.74)	0.729	0.49 (0.13–1.80)	0.282	0.44 (0.11–1.73)	0.240
Back pain (backache)	0.74 (0.37–1.47)	0.393	0.80 (0.38–1.70)	0.562	0.79 (0.40–1.55)	0.490
Rheumatologic disease	0.40 (0.19–0.84)	**0.015**	0.88 (0.40–1.93)	0.742	0.63 (0.30–1.34)	0.230
Depression	0.50 (0.23–1.09)	**0.081**	0.32 (0.13–0.75)	**0.009**	0.15 (0.06–0.40)	**< 0.001**
Other pathologies						
Back	1 (0.57–1.75)	0.992	1.15 (0.63–2.08)	0.647	0.82 (0.48–1.40)	0.460
Homolateral hip	0.62 (0.20–1.89)	0.402	0.28 (0.08-1)	0.050	0.53 (0.17–1.60)	0.259
Contraletral hip	1.01 (0.39–2.60)	0.982	0.44 (0.15–1.25)	0.122	0.68 (0.27–1.76)	0.427
Contralateral knee	0.87 (0.56–1.35)	0.535	0.70 (0.44–1.12)	0.134	1.05 (0.69–1.60)	0.833
Upper limbs	0.91 (0.33–2.51)	0.859	1 (0.33–3.04)	0.995	1.29 (0.49–3.40)	0.606
OA severity (Ahlbäck scale)						
Mild or moderate	-ref-	-ref-	-ref-	-ref-	-ref-	-ref-
Severe	1.22 (0.70–2.14)	0.485	1.84 (0.97–3.50)	0.062	2.53 (1.35–4.73)	**0.004**
Days in hospital	0.98 (0.92–1.03)	0.419	0.96 (0.90–1.03)	0.252	0.96 (0.91–1.02)	0.186
Postoperative complications	1.31 (0.70–2.46)	0.404	1.98 (0.99–3.95)	0.054	1.50 (0.82–2.73)	0.189
Readmissions (6 months)	0.54 (0.30–0.98)	**0.042**	0.58 (0.30–1.11)	0.101	0.60 (0.32–1.12)	0.110
Complications from hospital discharge	0.35 (0.16–0.76)	**0.007**	0.25 (0.11–0.59)	**0.002**	0.51 (0.23–1.12)	0.093
Rehabilitation from hospital discharge	1.38 (0.86–2.22)	0.181	1.64 (0.99–2.73)	0.057	1.37 (0.87–2.16)	0.174
Reintervention during the follow-up	0.56 (0.28–1.11)	0.095	0.49 (0.23–1.04)	0.062	0.35 (0.16–0.79)	**0.011**

Abbreviations: WOMAC, Western Ontario and McMaster Universities Osteoarthritis Index (0, best; 100, worst); BMI = Body Mass Index; OA = Osteoarthritis; CI, Confidence interval; MCID, Minimal clinically important difference; ref: reference group

This logistic regression models have been adjusted for the corresponding WOMAC baseline scores

^*^Changes in WOMAC exceeding the cut-offs points of the MCID (Pain = 29.88; Stiffness = 26.76; Function = 32.72)

^**^An Odds ratio < 1 indicates a lower likelihood of exceeding minimal clinically important difference; an Odds ratio > 1 indicates a higher likelihood of exceeding minimal clinically important difference

**Table 5:** Factors associated jointly with change in WOMAC at 10 years exceeding minimal clinically important difference: multivariable analysis

Factors	Change in WOMAC exceeding MCID^*^							
	Pain			Stiffness		Function		
	Odds ratio^**^ (95% CI)	*p* *value*	Odds ratio (95% CI)		*p* *value*	Odds ratio (95% CI)	*p* *value*	
Baseline WOMAC	1.06 (1.04–1.08)	< 0.001	1.07 (1.06–1.09)		< 0.001	1.06 (1.04–1.08)	< 0.001	
Age (continuous)						0.94 (0.90–0.97)	0.001	
BMI categorized								
BMI < 25			-ref-		-ref-	-ref-	-ref-	
25 ≤ BMI < 30			0.19 (0.05–0.66)		0.009	0.52 (0.21–1.29)	0.159	
30 ≤ BMI < 35			0.18 (0.05–0.66)		0.009	0.35 (0.14–0.88)	0.026	
BMI ≥ 35			0.20 (0.05–0.79)		0.022	0.25 (0.08–0.74)	0.012	
Comorbidity								
Peripheral vascular disease						0.49 (0.24–0.99)	0.048	
Depression	0.42 (0.18–0.94)	0.036	0.26 (0.09–0.77)		0.015	0.19 (0.06–0.56)	0.003	
Rheumatologic disease	0.37 (0.17–0.80)	0.011						
Readmissions (6 months)						0.41 (0.18–0.94)	0.035	
Complications from hospital discharge			0.31 (0.11–0.91)		0.032			
Rehabilitation from hospital discharge			2.12 (1.18–3.80)		0.012			
**AUC (95%CI)**	0.74 (0.69–0.79)			0.85 (0.81–0.89)		0.78 (0.73–0.82)		

Abreviations: BMI, Body Mass Index; WOMAC, Westen Ontario and McMaster Universities Osteoarthritis Index (0, best; 100, worst); CI, Confidence interval; MCID, Minimal clinically important difference; AUC: Area under the ROC curve; ref: reference group

^*^Changes in WOMAC exceeding the cut-offs points of the MCID (Pain = 29.88; Stiffness = 26.76; Function = 32.72)

^**^An Odds ratio < 1 indicates a lower likelihood of exceeding minimal clinically important difference; an Odds ratio > 1 indicates a higher likelihood of exceeding minimal clinically important difference

These models have been adjusted for age and gender and they only appear in the table if some of them were statistically significant

### Discussion

In this study, we have analyzed changes in the HRQOL at 10 years after TKA and compared it with general population. Additionally, we have identified variables most closely associated with greater improvements or with the probability of exceeding the MCID. To our knowledge, only a very few previous studies have analyzed HRQOL in patients with OA over ten years after TKA. One of the strengths of our study compared to these other studies was that we had a larger sample size and only included patients with knee OA. Specifically, Nuñez et al. [6] analyzed HRQOL at 7 years in 112 patients who had undergone TKA in Spain; Rat et al. [13] described HRQOL at 10 years in 89 patients who had undergone total knee or hip arthroplasty in France; and Bourne et al. [14] analyzed it in a 728 patients with OA, inflammatory arthritis and other diagnoses at 11 years. We included 471 patients, notably more than the first two studies, all had undergone TKA and only patients with OA were included. This is an important point since it has been suggested that HRQOL could differ with the conditions (i.e. OA vs. rheumatoid arthritis) [4]. Another strength is that we have analyzed which preoperative factors were associated with a greater probability of exceeding the MCID, unlike the other studies. Therefore, our study provides important novel information about which patients may benefit the most from the intervention in the long term, and our data are very useful for guiding patient care.

Ten years after surgery, we found that patients’ HRQOL was better than before surgery in all WOMAC dimensions and from 6 months to 10-years patients’ scores remained stable, except in function, for which scores worsened, though the changes were small. Patterns in the MCID results are similar. These results, like previous studies, confirm that functional abilities are impaired several years after TKA [13, 28, 29]. This reflects a natural age-related decline due to an increasing number of comorbidities, multiple sites of osteoarthritis and therefore, patients’ loss of physical function [29].

Scores for all of the SF-36 subscales 10 years after TKA were lower than those of the general population. These results are along the same lines as findings of Rat et al. [13] who found that 10 years after TKA scores for all SF-36 dimensions were lower than those for the reference population. The authors concluded that after TKA, impaired HRQOL persist over time despite substantial improvement in condition.

We observed that patients who were older, with higher BMIs and/or depression at baseline gain less in HRQOL measured by WOMAC at 10 years. Further, patients with a rheumatic disease gain less in pain and patients with readmissions at 6 months gain less in function. Preoperative HRQOL scores were also predictive of improvements in HRQOL 10 years after surgery. Rat et al. [13], found that sociodemographic factors were not associated with HRQOL after surgery. In contrast, Nuñez et al. [6] found, as in our study, that female sex, severe and morbid obesity and clinical factors such as some self-reported complications did negatively influence changes in WOMAC scores.

Although Nuñez et al. [6] found that women had significantly worse WOMAC scores at 7 years, reports vary in this respect. Perhaps this may be due in part to the fact that Nuñez et al. [6], as in most similar studies, used only postoperative scores rather than pre- to postoperative changes in scores. Another possible explanation would be that women in our study had a poorer HRQOL at baseline, but we have adjusted our results for baseline scores.

Previous short- and medium-term follow-up studies have also yielded inconsistent findings about the role of sociodemographic and clinical characteristics in terms of which are associated with better outcome. Regarding BMI, the prevailing opinion has been that high BMI has a negative impact on HRQOL after TKA [7, 30, 31].

In the case of age, Schilling et al. [32] in a study with TKA patients found that the age was a predictor of gains in HRQOL at seven years. This could be due to the fact that older age is frequently associated with more comorbid conditions, greater frailty, physiological loss of functions and, in some cases, the onset of sarcopenia, differences which may result in poorer outcomes [11, 32–34]. Previous studies, like ours, have found that comorbid conditions, specially, chronic musculoskeletal pain not related to knee OA, are associated with worsening physical function and pain [35, 36]. Rheumatic diseases are a major source of pain as are other comorbid diseases such as fibromyalgia, low back pain and polyarthritis and these have been shown to have a substantial independent impact on HRQOL outcomes after TKA [7, 37, 38]. These factors suggest that treatment should address not only the affected knee but also other joints to improve outcomes.

Regarding depression, the results are more conflicting. In one study [39], depression was related with less optimal improvement in knee function at 2 years, while in another [40] depressed patients had similar improvements in HRQOL to non-depressed patients. One of the main reasons for this discrepancy could be that these concepts are measured with many different questionnaires. Nonetheless, patients with preoperative depression complained of more pain after surgery and used more postoperative resources [41]. Identification and treatment of depression before surgery may therefore be another important strategy to improve outcomes after TKA surgery [41, 42].

To assess which patients benefited the most from the intervention in the long term, we also considered latent variables that might influence TKA outcomes in the long term in terms of whether patients achieve MCIDs. We found the same variables as those previously observed to be associated with gains in HRQOL. Nevertheless, age only seems to be associated with exceeding the MCID in WOMAC function. On the other hand, the MCID analysis identified other variables related to exceeding the MCID: having peripheral vascular disease, and both complications and rehabilitation after discharge. Specifically, these new data suggested that patients with peripheral vascular disease, with complications after discharge and without rehabilitation after discharge and those with the characteristics listed above are less likely to achieve the MCID in some of the WOMAC dimensions.

Patients with peripheral vascular disease before surgery were less likely to exceed the MCID and this could be, in part, because they tend to have more complications after surgery, especially vascular complications [43]. The rate of vascular complications following TKA may be as high as 25% in patients with pre-existing peripheral vascular disease and such complications have serious consequences [43]. Patients at risk of developing vascular complications should be identified and assessed pre-operatively in order to minimize the incidence of these complications.

Regarding patients who develop complications after hospital discharge being less likely to achieve the MCID, a number of complications related to the prosthesis may result in prosthetic failure and revision. Although these factors appear to have little impact on the short-term outcomes, they might have greater influence on the longevity of the implant itself [11], and in turn, this could lead to a poorer HRQOL in the long term [44].

Study findings must be interpreted considering the study limitations. Firstly, the losses in the follow-up of the baseline cohorts should be noted. However, losses in the follow-up are common in long-term follow-up studies, even more so when dealing with older patients. Further, patients who did not respond to the questionnaire sent at 10 years after the TKA surgery had worse preoperative scores in function. However, although these differences were statistically significant, these patients do not seem to represent a group with markedly more severe impairment than responders, given the magnitude of the differences in points between the two groups. Another limitation was that in this study we did not have a generic tool to measure quality of life at baseline, at 6 months and at 10 years. Besides, despite the fact that 10-year follow-up data require such a time period, the selection of a cohort recruited between 2002 and 2004 (18 and 20 years) and between 2005 and 2006 (16 and 17 years) provides a time lag of 6 to 10 years. Changes in TKA surgery techniques and enhance recovery programmes may have impacted on outcomes. However, we did not adjust for such factors in our analysis. Furthermore, given the differences in surgical and perioperative management, this may question the external validity of these findings to contemporary practice. This should be considered when interpreting these findings. Another limitation of our study would be that we have not captured the implant failure as a possible confounding variable. The challenge in assessing 10 year data for TKA is that we are in the realms of revision and implant loosing/failure. Consequently, the HRQOL may be a function for some people, of a failing implant. Therefore, further studies are needed to examine whether other factors are influencing HRQOL at 10 years. Finally, the percentages of variance explained in linear regression ranged from 22.8 to 39.4%. Similar studies had similar results to ours such as the study carried out by Nuñez et al. [6] whose percentage of variance explained was even lower than ours (from 14 to 33%). However, we believe that some other non-registered parameters may also influence changes in HRQOL.

To our knowledge, this is one of the few prospective long-term follow-up studies focusing on factors associated with gains in HRQOL. We identified factors that negatively influence gains in HRQOL in the long term and that it could be minimized with better management. Further, our findings have other implications for patient care. Identifying which patients would benefit most in the long term from TKA should help surgeons to better inform patients about the likely outcomes of this type of intervention. Moreover, information on factors related to gains in HRQOL after TKA might help us meet patient needs, and treatment to address risk factors such as depression, rheumatic disease and complications after hospital discharge could have a great impact on changes in HRQOL in the long term.

## Conclusions

In brief, based on our data, we conclude that low preoperative scores in each dimension, being elderly and/or obese, having certain comorbidities, and being readmitted and/or developing complications after hospital discharge (6 months) are good predictors of smaller gains in HRQOL.

## Electronic supplementary material

Below is the link to the electronic supplementary material.

Supplementary Material 1

Supplementary Material 2

Supplementary Material 3

Supplementary Material 4

## Data Availability

The datasets used and analyzed during the current study are available from the corresponding author on reasonable request.

## List of Abbreviations

OA: Osteoarthritis
HRQOL: Health related quality of life
TKA: Total knee arthroplasty
MCID: minimal clinically important difference
WOMAC: Western Ontario and McMaster Universities Arthritis Index
SF-36: 36-item short form health survey
BMI: Body mass index
SD: Standard deviation
ES: Standardized effect sizes
AUC: Area under curve.
