# Supplementary material for: Long-term health related quality of life in total knee arthroplasty
Source: BMC Musculoskelet Disord. 2023 Apr 25;24:327. doi: 10.1186/s12891-023-06399-6 (PMC10127408; doi:10.1186/s12891-023-06399-6)
Supplement: Supplementary file 1 — Supplementary Material 1 [file 12891_2023_6399_MOESM1_ESM.docx]

**Table 1.** Baseline characteristics of responders s to the long-term follow-up and the excluded patients due to death.

| **Variables** | **Responders**  (n=471) | **Patients excluded due to death**  (n=246) | **p-value** |
| --- | --- | --- | --- |
| **Age** in years: mean (SD) | 69.73 (6.26) | 74.55 (6.13) | <0.0001 |
| **Age categorized:** n (%) |  |  | <0.0001 |
| ≤ 65 | 102 (22.67) | 20 (8.51) |  |
| 65-75 | 263 (58.44) | 80 (34.04) |  |
| ≥ 75 | 85 (18.89) | 135 (57.45) |  |
| **Gender**: female: n (%) | 355 (75.37) | 145 (58.94) | <0.0001 |
| **BMI:** mean (SD) | 30.22 (4.34) | 29.72 (4.51) | 0.1618 |
| **BMI categorized:** n (%) |  |  | 0.1078 |
| BMI < 25 | 38 (8.78) | 35 (15.02) |  |
| 25 ≤ BMI < 30 | 181 (41.80) | 90 (38.63) |  |
| 30 ≤ BMI < 35 | 159 (36.72) | 81 (34.76) |  |
| BMI ≥ 35 | 55 (12.60) | 27 (11.59) |  |
| **Having social support:** n (%) | 397 (87.83) | 197 (83.83) | 0.1458 |
| **Civil status:** n (%) |  |  | 0.0603 |
| Married | 290 (62.91) | 132 (54.77) |  |
| Partner | 25 (5.42) | 10 (4.15) |  |
| Divorced | 5 (1.08) | 2 (0.83) |  |
| Widowed | 128 (27.77) | 82 (34.02) |  |
| Single | 13 (2.82) | 15 (6.22) |  |
| **Comorbidity:** n (%) |  |  |  |
| Myocardial infarction | 14 (3.14) | 19 (7.98) | 0.0049 |
| Congestive heart disease | 17 (3.80) | 21 (8.82) | 0.0063 |
| Hypertension | 243 (54.61) | 145 (61.18) | 0.0988 |
| Peripheral vascular disease | 62 (13.90) | 33 (13.87) | 0.9897 |
| Chronic pulmonary disease | 38 (8.52) | 25 (10.50) | 0.3927 |
| Ulcer disease | 17 (3.81) | 11 (4.62) | 0.6105 |
| Mild liver disease | 3 (0.67) | 5 (2.10) | 0.1344 |
| Moderate or severe chronic kidney disease | 2 (0.45) | 3 (1.26) | 0.3483 |
| Diabetes | 52 (11.69) | 40 (16.81) | 0.0618 |
| Cancer Tumour | 6 (1.35) | 8 (3.36) | 0.0912 |
| Cerebrovascular disease | 13 (2.91) | 30 (12.61) | 0.0558 |
| Back pain (backache) | 44 (9.87) | 17 (7.14) | 0.0101 |
| Rheumatologic disease | 38 (8.52) | 39 (16.39) | 0.0129 |
| Connective tissue disease | 2 (0.45) | 20 (8.40) | 0.9583 |
| Depression | 36 (8.07) | 1 (0.42) | 1 |
| **Other pathologies:** n (%) |  |  |  |
| Back | 79 (19.13) | 58 (25.55) | 0.0581 |
| Homolateral hip | 17 (4.17) | 17 (7.49) | 0.0747 |
| Contraletral hip | 23 (5.62) | 22 (9.69) | 0.0552 |
| Contralateral knee | 205 (49.52) | 128 (55.90) | 0.1211 |
| Upper limbs | 20 (4.91) | 20 (8.81) | 0.0530 |
| **OA severity** (Ahlbäck scale): n (%) |  |  | 0.1363 |
| Mild | 6 (1.79) | 1 (0.52) |  |
| Moderate | 75 (22.32) | 32 (16.49) |  |
| Severe | 255 (75.89) | 161 (82.99) |  |
| **Surgical risk** (ASA): n (%) |  |  | 0.0084 |
| Low (ASA I, II, III) | 426 (99.07) | 228 (95.80) |  |
| High (ASA IV) | 4 (0.93) | 10 (4.20) |  |
| **Intraoperative complications:** n (%) | 8 (1.87) | 3 (1.27) | 0.7545 |
| **Postoperative complications:** n (%) | 61 (14.49) | 44 (18.64) | 0.1632 |
| **Readmissions (6 months)**: n (%) | 62 (14.00) | 38 (16.24) | 0.4339 |
| **Days in hospital:** median (IQR) | 12 (10 – 15) | 13.56 (10 – 15) | 0.2029 |
| **Complications from hospital discharge:** n (%) | 37 (8.55) | 29 (12.50) | 0.1040 |
| **Rehabilitation from hospital discharge:** n (%) | 157 (41.10) | 63 (30.88) | 0.0150 |

Abbreviations: SD, Standard deviation; IQR, Interquartile range; OA, Osteoarthritis; BMI, Body Mass Index.
