# Supplementary material for: Long-term health related quality of life in total knee arthroplasty
Source: BMC Musculoskelet Disord. 2023 Apr 25;24:327. doi: 10.1186/s12891-023-06399-6 (PMC10127408; doi:10.1186/s12891-023-06399-6)
Supplement: Supplementary file 2 — Supplementary Material 2 [file 12891_2023_6399_MOESM2_ESM.docx]

**Table 2.** Follow-up characteristics of responders to the long-term follow-up at 10 years

| **Variables** | Characteristics at 10 years |
| --- | --- |
|  |  |
| **Age** in years: mean (SD) | 79.26 (6.58) |
| **Age categorized:** n (%) |  |
| ≤ 65 | 14 (2.97) |
| 65-75 | 91 (19.32) |
| ≥ 75 | 366 (77.71) |
| **BMI:** mean (SD) | 29.92 (4.91) |
| **BMI categorized:** n (%) |  |
| BMI < 25 | 63 (13.85) |
| 25 ≤ BMI < 30 | 186 (40.88) |
| 30 ≤ BMI < 35 | 153 (33.63) |
| BMI ≥ 35 | 53 (11.65) |
| **Civil status:** n (%) |  |
| Married / Partner | 233 (50.43) |
| Divorced | 7 (1.52) |
| Widowed | 209 (45.24) |
| Single | 13 (2.81) |
| **Comorbidity:** n (%) |  |
| Myocardial infarction | 22 (4.72) |
| Congestive heart disease | 47 (10.09) |
| Peripheral vascular disease | 21 (4.51) |
| Chronic pulmonary disease | 55 (11.80) |
| Ulcer disease | 1 (0.21) |
| Liver disease | 2 (0.45) |
| Moderate or severe chronic kidney disease | 1 (0.21) |
| Diabetes | 131 (28.11) |
| Cancer Tumour | 67 (14.38) |
| Cerebrovascular disease | 39 (8.37) |
| Connective tissue disease | 3 (0.64) |
| Dementia | 29 (6.22) |
| **Other pathologies:** n (%) |  |
| Back | 221 (47.63) |
| Homolateral hip | 51 (11.02) |
| Contraletral hip | 62 (13.39) |
| Contralateral knee | 192 (41.38) |
| **Knee reintervention**: n (%) | 43 (9.25) |
| **Reason for reintervention**, n (%) |  |
| Infection | 6 (13.95) |
| Aseptic loosening / Mobilization | 16 (37.21) |
| Instability | 5 (11.63) |
| Fracture | 1 (2.33) |
| Pain | 10 (23.26) |
| Implant failure | 8 (18.60) |
| Error / Technical problems | 1 (2.33) |
| **Time from intervention to reintervention**  (years), mean (SD) | 3.10 (3.03) |

Abbreviations: SD, Standard deviation; BMI, Body Mass Index.
