# Supplementary material for: Long-term health related quality of life in total knee arthroplasty
Source: BMC Musculoskelet Disord. 2023 Apr 25;24:327. doi: 10.1186/s12891-023-06399-6 (PMC10127408; doi:10.1186/s12891-023-06399-6)
Supplement: Supplementary file 3 — Supplementary Material 3 [file 12891_2023_6399_MOESM3_ESM.docx]

**Additional figure 1.** Western Ontario and McMaster Universities Osteoarthritis Index (WOMAC) scores at pre-intervention, 6 months and 10 years after total knee arthroplasty. WOMAC: 0, best; 100, worst.
