# Supplementary figures and images for: Long-term health related quality of life in total knee arthroplasty
Source: BMC Musculoskelet Disord. 2023 Apr 25;24:327. doi: 10.1186/s12891-023-06399-6 (PMC10127408; doi:10.1186/s12891-023-06399-6)

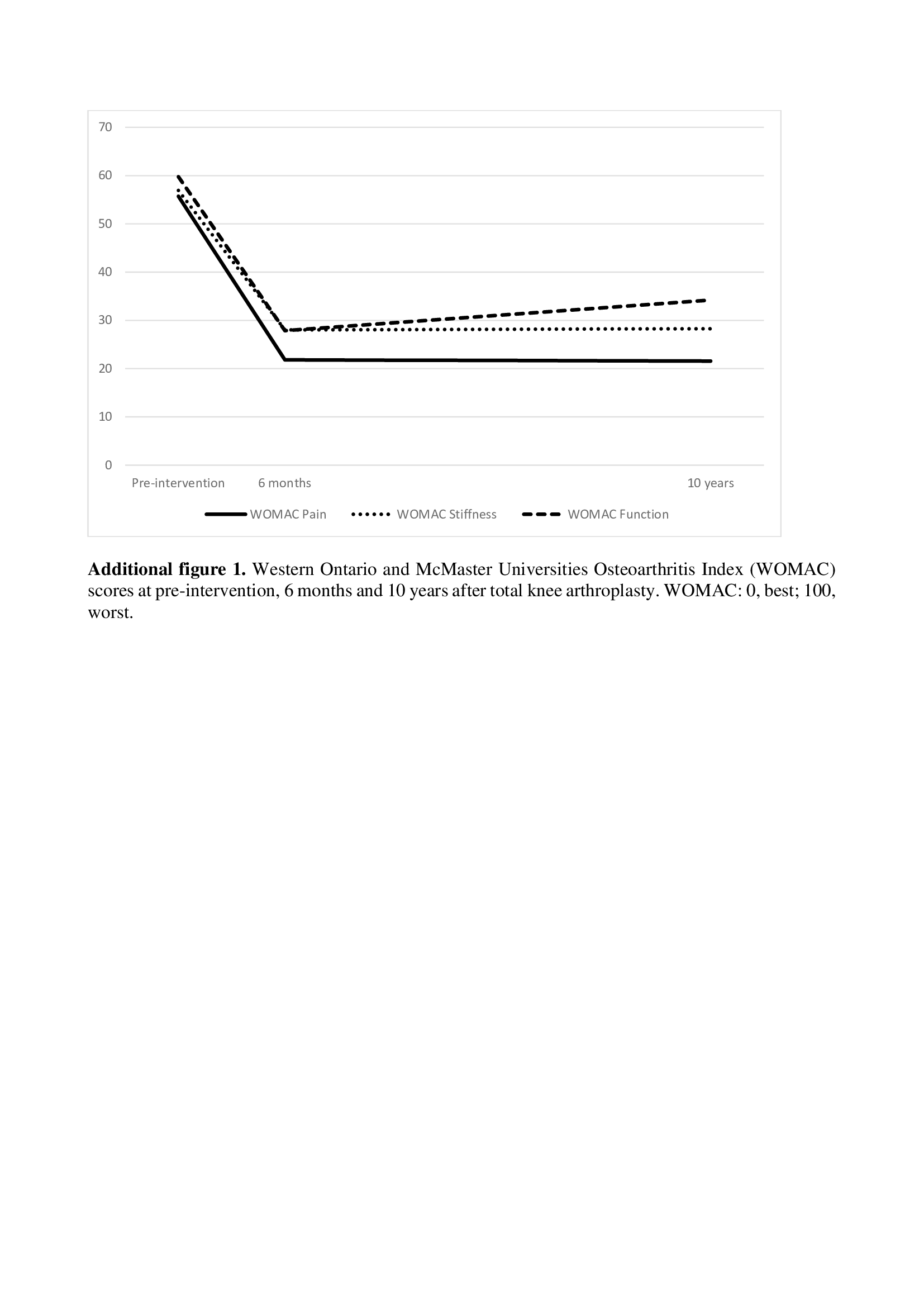

Supplement: Supplementary file 4 — Supplementary Material 4 [file 12891_2023_6399_MOESM4_ESM.png]
